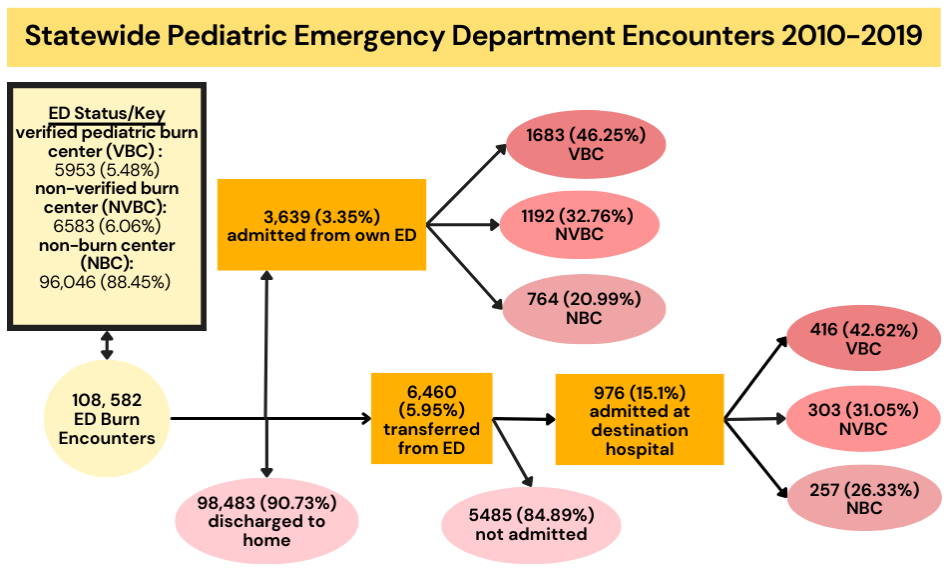# 107 Patterns and Disparities in the Triage of Pediatric Burns

**DOI:** 10.1093/jbcr/iraf019.107

**Published:** 2025-04-01

**Authors:** Claire Justin, Eloise Stanton, Clifford Sheckter

**Affiliations:** Santa Clara Valley Medical Center; University of Southern California; Santa Clara Valley Medical Center

## Abstract

**Introduction:**

The American Burn Association (ABA) verifies pediatric burns centers to maintain the highest quality in the care of burned children. However, children may be triaged to other facilities. To date, there are no population health level studies exploring factors that determine which burned children are treated at pediatric verified burn centers. Further, there is a need to define characteristics of those most at risk for under-referral. We hypothesize that factors including race, distance to the burn center, social deprivation index (SDI), and insurance status will impact treatment at verified pediatric burn centers.

**Methods:**

A state’s health care information database was queried from 2010-2019 extracting all pediatric emergency department (ED) and inpatient admissions. Individual patients were linked between ED and inpatient encounters. The distance between the burn center and patient’s home was calculated by using the geographic coordinates of the patient’s and facility’s zip codes. Facilities were categorized as pediatric verified burn centers, non-verified burn centers, or non-burn centers using information from the ABA. The primary outcome was admission to a verified pediatric burn center which was modeled with multivariable logistic regression. Study variables included race, social deprivation index (SDI), insurance status, and travel distance. The model was adjusted for age, TBSA, non-accidental trauma, and inhalation injury.

**Results:**

There were 108,541 ED visits of which 10,099 (9.3%) were admitted or transferred. Of patients admitted from an ED, 45.48 % were admitted to pediatric verified centers, 32.39% were admitted to non-verified burn centers, and 22.12% were admitted to non-burn centers. 5.95% were transferred from initial ED to a different facility. Of all admitted transfers, 42.62% occurred to verified centers compared to 31.05% to non-verified centers and 26.33% to non burn centers. SDI (aOR 0.99, 95% CI 0.99-0.99, p = 0.002) and public health insurance status (aOR 0.50, CI 0.41- 0.59, p< 0.001) were significantly associated with admission to verified centers.

**Conclusions:**

Children living in areas with higher SDI or with public health insurance were less likely to be admitted to a verified pediatric burn center from the ED. The data suggests a significant economic disparity in access to specialized burn care. Future interventions can be targeted to address the link between childhood poverty and poorer access to quality burn care.

**Applicability of Research to Practice:**

Understanding referral patterns in pediatric burn patients is crucial for shaping local and statewide efforts to improve access to specialized, high-quality care for all children with burns. Identifying barriers to referral can guide revisions to referral guidelines and enhance the effectiveness of American Burn Association educational initiatives, ultimately ensuring more equitable access to pediatric-specific burn care.

**Funding for the Study:**

Institutional Pediatrics Residency Research Grant - January 2024